# Scientific use of the finite element method in Orthodontics

**DOI:** 10.1590/2176-9451.20.2.119-125.sar

**Published:** 2015

**Authors:** Luegya Knop, Luiz Gonzaga Gandini, Ricardo Lima Shintcovsk, Marcia Regina Elisa Aparecida Schiavon Gandini

**Affiliations:** 1 PhD resident in Orthodontics, Universidade Estadual Paulista (UNESP), School of Dentistry, Department of Orthodontics and Pediatric Dentistry, Araraquara, São Paulo, Brazil; 2 Assistant professor, Universidade Estadual Paulista (UNESP), School of Dentistry, Department of Orthodontics and Pediatric Dentistry, Araraquara, São Paulo, Brazil; 3 Volunteer professor, Universidade Estadual Paulista (UNESP), School of Dentistry, Department of Orthodontics and Pediatric Dentistry, Araraquara, São Paulo, Brazil

**Keywords:** Bioengineering, Finite element method, Orthodontics

## Abstract

**INTRODUCTION:**

The finite element method (FEM) is an engineering resource applied to calculate the stress and deformation of complex structures, and has been widely used in orthodontic research. With the advantage of being a non-invasive and accurate method that provides quantitative and detailed data on the physiological reactions possible to occur in tissues, applying the FEM can anticipate the visualization of these tissue responses through the observation of areas of stress created from applied orthodontic mechanics.

**OBJECTIVE:**

This article aims at reviewing and discussing the stages of the finite element method application and its applicability in Orthodontics.

**RESULTS:**

FEM is able to evaluate the stress distribution at the interface between periodontal ligament and alveolar bone, and the shifting trend in various types of tooth movement when using different types of orthodontic devices. Therefore, it is necessary to know specific software for this purpose.

**CONCLUSIONS:**

FEM is an important experimental method to answer questions about tooth movement, overcoming the disadvantages of other experimental methods.

## Abstract (pt)

**INTRODUÇÃO:**

o Método de Elementos Finitos (MEF) é um recurso da Engenharia empregado para calcular o estresse e a deformação de estruturas complexas, e tem sido amplamente utilizado nas pesquisas em Ortodontia. Apresenta a vantagem de ser um método não-invasivo e preciso, que fornece dados quantitativos e detalhados acerca das reações fisiológicas que podem ocorrer nos tecidos.

**OBJETIVO:**

esse artigo pretende realizar uma revisão da literatura sobre as etapas para realização do Método de Elementos Finitos, bem como de sua aplicabilidade na Ortodontia.

**RESULTADOS:**

o MEF é capaz de avaliar a distribuição do estresse na interface entre o ligamento periodontal e o osso alveolar, bem como a tendência de deslocamento em diversos tipos de movimentos dentários, quando utilizados diferentes tipos de aparelhos. Para tanto, é necessário conhecimento de *softwares* específicos para esse fim.

**CONCLUSÕES:**

o MEF é um importante método experimental que pode esclarecer questionamentos acerca da movimentação dentária, superando as desvantagens de outros métodos experimentais.

## INTRODUCTION

Doubts and questions on the use of the finite element method (FEM) for health researches, especially regarding Dentistry and Orthodontics, are very frequent. In this context, we present this special topic to elucidate concepts, principles, objectives and application of this method as part of a line of research included in the postgraduate studies of the School of Dentistry of Universidade Estadual Paulista (UNESP) - Araraquara.

The FEM is an engineering resource used to calculate stress and deformations in complex structures, and it has been widely applied in biomedical research.^1^^,^ 2

On the scope of Structural Engineering, the use of the FEM aims at establishing the state of tension and deformation of an arbitrary-geometry solid submitted to exterior actions. This type of calculation has the generic designation of analyzing structures, and it is common in studies on buildings, bridges, dams, etc. When a structure is required to be projected, it is common to proceed with a succession of analyses and alterations of its characteristics in order to reach a satisfactory solution regarding either the economy or the verification of functional and regulatory requirements.^3^

According to Azevedo,^3^ Ray Clough is the author of the oldest written record using the designation "finite element", in 1960; a few other techniques previously known had been incorporated into the FEM. The 60s and the early 70s were the landmark of the major steps towards the FEM development that directed the method to reach its current widely accepted format. 

By applying the FEM, Orthodontics is able to shape and analyze any material or dentomaxillofacial structures.^4^

The FEM principle is based on the division of a complex structure into smaller sections called elements^5^ in which physical properties, such as the modulus of elasticity, are applied to indicate the object response against an external stimulus such as an orthodontic force. It represents a great advantage of the method, since the degree of simplification can be controlled.^6^

## LITERATURE REVIEW AND DISCUSSION

Several studies on orthodontic-force-induced tooth movement were conducted using experimental animal models.^7^^-^ 11 These studies provide indications on the consequences of applying orthodontic forces to human tissues.^6^ Since this type of experiment requires the use of living animals in laboratory, it is frequent that ethics committees on animal research have objections. With FEM, it is possible to anticipate the tissue responses to orthodontic mechanics applied. Alternative experimental models used to analyze the biomechanics of tooth movement include photoelastic models;^12^ however, they have the disadvantage of exploring only the surface of the model, leaving internal structures, such as the periodontal ligament, behind.

For overcoming the aforementioned disadvantages, the FEM has reformed biomechanical research in Orthodontics. It represents a non-invasive, accurate method that provides quantitative and detailed data regarding the physiological responses occurring in tissues, such as the periodontal ligament and the alveolar bone.^13^ According to Middleton et al,^14^ this accurate analysis of potential stress and tension occurring in tooth tissues is difficult to be obtained through any other experimental technique due to the interaction between surrounding tissues and the individual response. Another advantage of the FEM is the possibility to study a homogenous sample while controlling all study variables.^2^

Several studies have investigated the action of orthodontic forces on the craniofacial complex using the FEM.^2^^,^ 13 ^,^ 15 ^-^ 20 For instance, the method enables the calculation of stress and deformation produced during the translation or distal tipping of an upper right canine in the exodontia area of a premolar.^2^

Mc Guinness et al^15^ applied the FEM to assess the distribution of orthodontic forces released by the Edgewise appliance. The authors used an upper canine bracket with slot 0.022-in, and a wire filling the slot. A force of 98.1 gF was applied exclusively on mesiodistal direction, parallel to the orthodontic wire. The authors observed that stress concentration was higher at the cervical margin of the periodontal ligament and on the tooth apex.

Kojima and Fukui^17^ sought to investigate possible orthodontic movements for anchorage teeth with the application of a passive BTP. The FEM results indicated that passive BTP presented almost no effect on anchorage maintenance due to the occurrence of mesial movement of molars when a mesial force was applied.

Tominaga et al^18^ proposed to analyze the en masse retraction in sliding mechanics. Their results demonstrated that when the hook is positioned between the lateral incisor and the canine using sliding mechanics, the en masse retraction of the anterior segment is more controlled.

Kanjanaouthaia et al^19^ employed the FEM to demonstrate that after having received force of 1 N in lingual direction, upper incisors that were more inclined presented higher concentration of stress on the apex when compared with incisors well positioned in buccolingual direction. 

To conduct this experimental method it is interesting to use a resource with anatomical records and modifications in CAD software so as to build geometrically superior and accurate models. To that purpose, it is necessary to build a virtual model using an image-processing and digital reconstruction software, such as Mimics (Materialize, Leuven Belgium) or Simpleware 4 (Simpleware Ltd., Exeter, United Kingdom). In general, regarding the maxillomandibular complex, these reconstructions are carried out through computed tomography.

Computed tomography should be obtained with cross-sections of at least 0.25 mm distance so as to achieve improved resolution. The sections will be recorded on DICOM format (Digital Imaging and Communications in Medicine) and imported into an image-processing and digital reconstruction software. The level of contrast and definition of clinical tomography lead to unsatisfactory results of the resources of automatic segmentation structures in the reconstruction software, which makes the limits of structures, such as the periodontal ligament, enamel or even the medullary and cortical bone, impossible to be established.

Micro CTs would enable the capture of details on gauge scale; however, the radiation dose emitted by micro CTs is above the limits recommended for humans, in addition to involving high costs and difficult access, which justifies the use of computed tomographies.^21^

**Figure 1 -:**
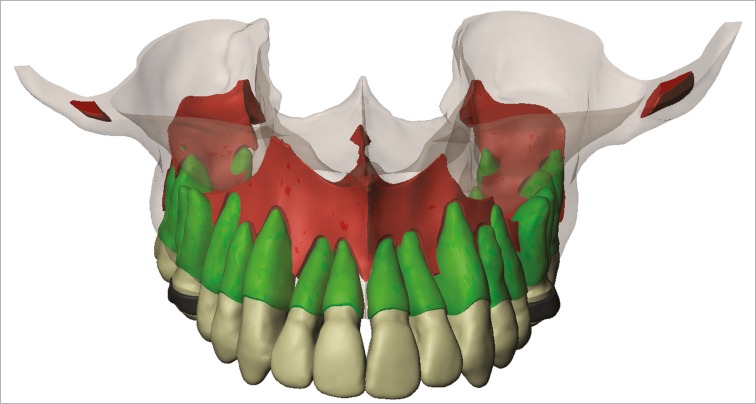
Virtual reconstruction of the maxilla by means of computed tomography.

Figure 1 illustrates a virtual model of the maxilla built through computed axial tomography (iCAT, Xoran Technologies, Ann Arbor, USA) totaling 218 sections of 640 x 640 voxels each.

The model presented a maxilla with 956,196 faces and each tooth with dozens of hundreds of polyhedral faces. Considering that, at this stage, the model had not been finished, we reduced the number of faces to a maximum of a few hundreds so as to carry out its edition. The simple reduction in the number of nonparametric faces leads to great distortion of the model, since the former are exclusively triangular and flat. In order to enable further edition without significant distortion, the models should be parameterized using Solidworks Premium software "scan to 3D" (Dassault Systemes, Solidworks Corps, USA), thereby making the transformation of nonparametric models into parametric models with NURBS faces (Non Uniform Rational Bases Splines), with minimum distortion, possible. Orthodontic components should also be virtually reconstructed with the aid of a digital calliper (Litz professional, Germany) and a digital microscope. 

The more structures are modelled, the more accurate are the results; however, it makes the model more difficult to be obtained and the analysis of the results more complex. Therefore, simpler models should be applied in order to obtain the same quantitative results. Modelling should be carefully assessed so as to simplify the model according to its actual needs and without compromising the results.^4^

In order to enable analysis by means of FEM, we also used the aforementioned software to conduct the transformation of the solid model into a mesh of bonds and elements, which is the discretization of the model. 

**Figure 2 -:**
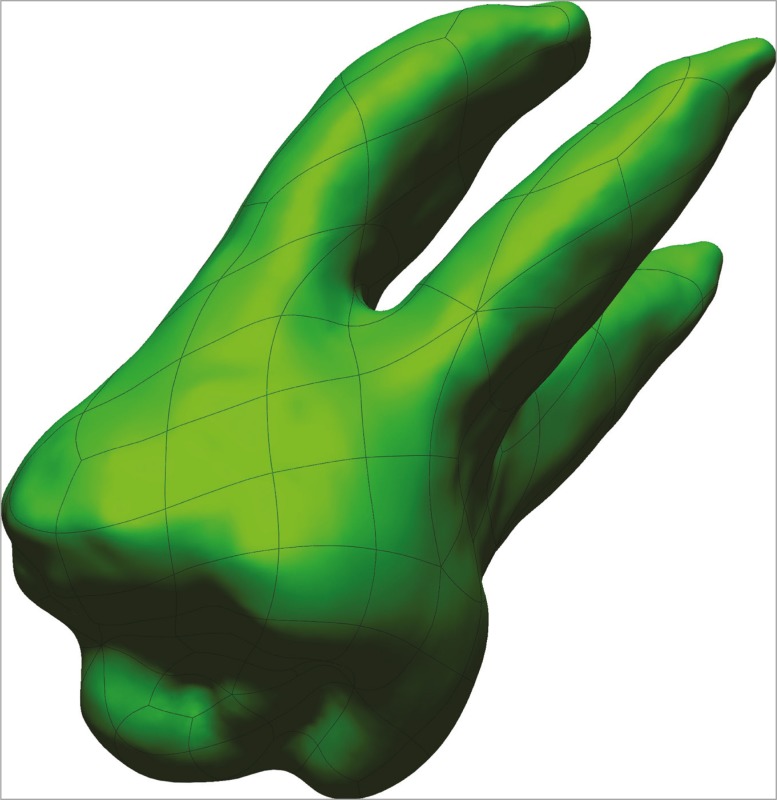
Example of elements and bonds of a molar. The bonds connect the elements to each other and are located in their extremities.

The elements represent coordinates in space and may present with several formats, in which case tetrahedrons and hexahedrons are the most common. The extremities of each element present points, or bonds, that connect the elements to each other forming an arranged mesh.^4^ It is the bonds that transmit information between elements (Fig 2).

To reach the ideal mesh of finite elements, we use a process of mesh refinement to verify the convergence of results with gradual increase in the number of bonds and elements until the difference of voltage peaks between mesh refinements is 5% or lower. These measures minimize the geometric error peculiar to a process of mesh discretization. 

After the entire virtual model is reconstructed and transformed into this mesh of finite elements, it is exported from Solidworks software to Ansys Workbench V11 software for finite elements simulation. (Ansys Inc., Canonsburg, PA, USA). This software requires the correct representation of the mechanical behavior of each component; thus, the model is set with a modulus of elasticity (Young) and Poisson's coefficient. Poisson's coefficient refers to the absolute value of the relationship between transverse and longitudinal deformations in an axial traction axis; whereas Young's model represents the inclination of the linear portion of (material) the stress-deformation diagram.^4^ Subsequently, we conduct the activation of the system with the application of charges using the aforementioned software. 

The results obtained by means of the FEM enable analysis of stress distribution produced by forces between the periodontal ligament and the bone, thereby demonstrating the areas of stress and, thus, the location where tooth movement occurs. It also enable us to infer about areas that are more prone to root resorption. These results are revealed by means of colors and arrows that are able to indicate the direction of tooth displacement after force application.

**Figure 3 -:**
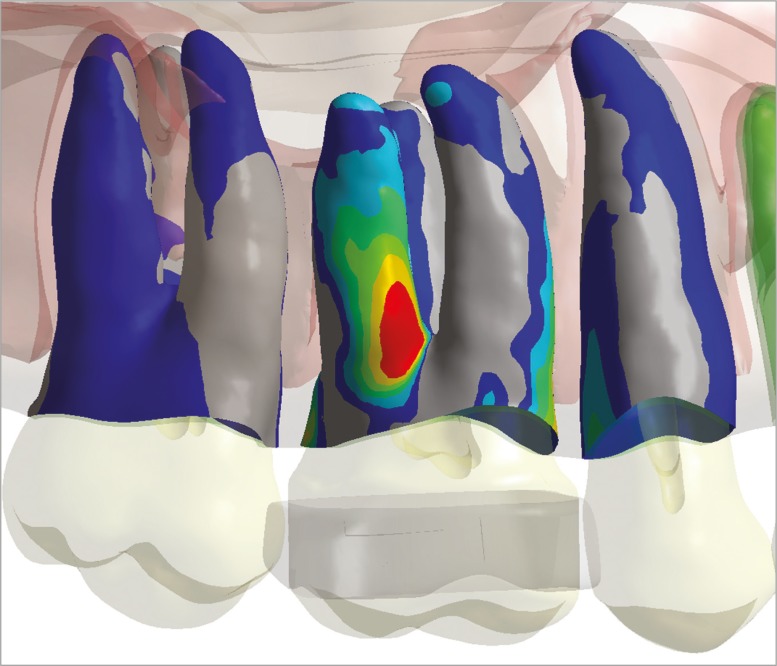
Areas of stress concentration on the periodontal ligament after submission to orthodontic force. Near the furcation area, we observe (red) higher stress concentration that gradually decreases towards the apex.

**Figure 4 -:**
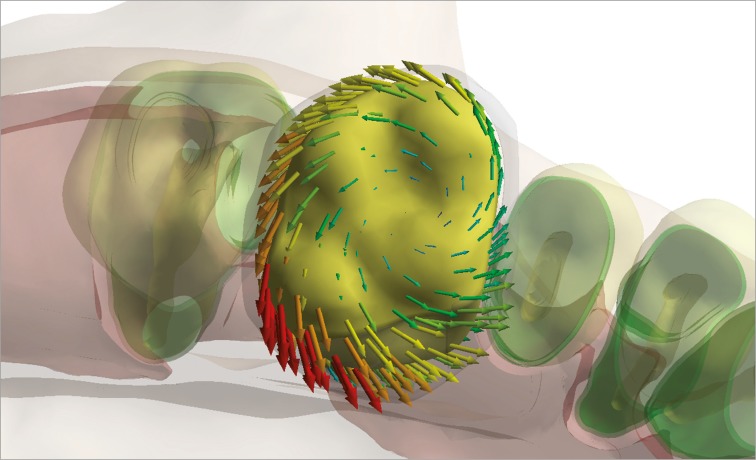
Arrows indicate the direction of tooth displacement and its intensity (red for higher displacement; green for lower displacement).

By verifying the colored illustration (Fig 3) of this experimental model, which is part of a research conducted at School of Dentistry/Unesp-Araraquara, we found that the region near the tooth fulcrum (red) holds a higher concentration of forces, and that the stress gradually decreases towards the apex (blue). In addition, using a resource of Ansys 14 Software, we assessed the tendency of movement to which the tooth is submitted against the application of the orthodontic force by means of colored arrows that demonstrate (red) the location with higher tooth displacement (Fig 4).

**Figure 5 -:**
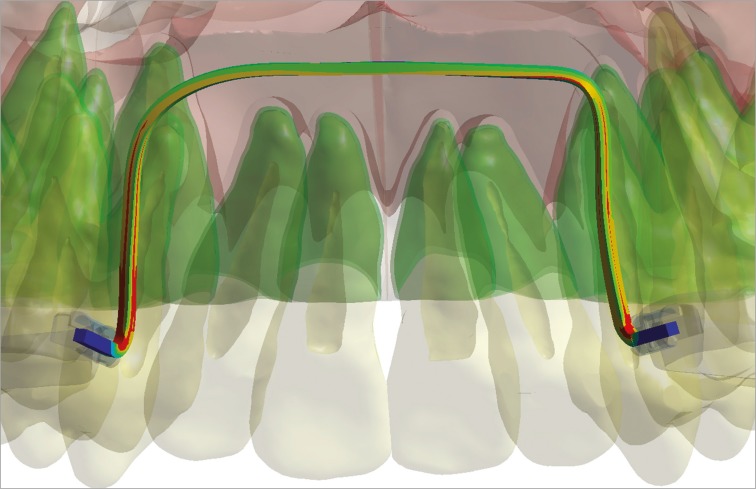
Areas of stress concentration on the orthodontic wire. Stress is more intense near the area with the wire bends (red).

In addition to viewing the stress distribution on the periodontal ligament, it is possible to observe the deformation of the orthodontic wire, and its region with higher stress concentration (Fig 5).

## FINAL CONSIDERATIONS

The Finite Element Method (FEM) proves to be an important instrument in orthodontic research, highlighting several points, such as: stress distribution areas in the periodontal ligament and alveolar bone during tooth movements; direction of the tooth displacement; the ideal position of orthodontic appliances during a specific mechanics; areas most likely to present root resorption; In addition the stress distribution on the archwires. MEF is able to overcome disadvantages of other experimental methods, as it is accurate, noninvasive, controls the study variables and provides quantitative data about internal structures of nasomaxillary complex, as the periodontal ligament. The method, however, requires knowledge in Computer Engineering, as it is run on very specific software.
